# ORGAN EXTRACTS AND THE DEVELOPMENT OF PSYCHIATRY: HORMONAL TREATMENTS AT THE MAUDSLEY HOSPITAL 1923–1938

**DOI:** 10.1002/jhbs.21548

**Published:** 2012-05-29

**Authors:** Bonnie Evans, Edgar Jones

## Abstract

The use of organ extracts to treat psychiatric disorder in the interwar period is an episode in the history of psychiatry which has largely been forgotten. An analysis of case-notes from The Maudsley Hospital from the period 1923–1938 shows that the prescription of extracts taken from animal testes, ovaries, thyroids, and other organs was widespread within this London Hospital. This article explores the way in which Maudsley doctors justified these treatments by tying together psychological theories of the unconscious with experimental data drawn from laboratory studies of human organs. It explores the logic behind these treatments and examines beliefs about their efficacy. The connection between this historical episode and current research in endocrinology and psychology is explored. © 2012 Wiley Periodicals, Inc.

In 1923, The Maudsley Hospital opened in London, United Kingdom, to pioneer new forms of treatment for mental illness. An ambitious agenda designed to research and treat severe psychiatric illness was drafted in 1907 by the mental scientist Henry Maudsley and Frederick Mott, a lecturer and physician at Charing Cross Hospital and director of the Central Pathological Laboratory of the London County Council (LCC) Asylums (Allderidge, 1991; Sharpey-Schafer, 2004; Jones, Rahman, & Woolven, 2007). Mott sought to model the Maudsley Hospital on the university psychiatric clinics of Munich, Berlin, and Heidelberg. He argued that intervention in the early stages of mental illness could prevent conditions such as hysteria, melancholia, and obsession from developing into severe and permanent mental disorder (Hayward, 2010, pp. 67–68). Critiques of the British asylum system can be traced back to the mid-nineteenth century but it was only in the early twentieth century that progressive physicians and administrators advanced workable alternatives based on German models. In 1911, a Committee on the Status of British Psychiatry was established following a series of debates over the implementation of a Diploma in Psychological Medicine. The Committee affirmed the need for early treatment and research centers and this view was supported by other physicians writing in the *Lancet* and the *British Medical Journal* (Hayward, 2010, pp. 68–69). In 1915, the Asylum's Committee of the LCC sought special powers from parliament so that the Maudsley could admit voluntary boarders, thereby avoiding the stigma of certification. This was granted although the disruption of the First World War meant that the Hospital did not open for the treatment of civilians until 1923, military patients having been admitted in January 1916.

The first medical superintendent, Edward Mapother, followed the principles laid down by Maudsley and Mott although he tempered some of their enthusiasm concerning the early treatment of severe forms of mental illness. Mapother was a former asylum doctor who had served in the Royal Army Medical Corps (RAMC) and had worked at the Maudsley in the immediate postwar period when it operated as a specialist treatment center for the Ministry of Pensions (Jones, 2010). As superintendent of the Maudsley from 1923, Mapother focused attention on acute and mild mental disorders. His long-term goal was to establish a multidisciplinary research facility, employing full-time scientists, and this, he believed, was the only way to understand severe mental illness. He died in 1940, just eight years before the establishment of the Institute of Psychiatry (IoP), whose laboratories were to achieve international recognition and which still serves as the leading British Institution for research in psychiatry, neuroscience, and clinical psychology.^1^ This article explores how the Maudsley's new approach to mental illness as a curable condition rather than as a life sentence stimulated experimental treatments. An analysis of patient case records has revealed that many of the Maudsley's first patients were treated with glandular extracts with the aim of increasing or redirecting their internal drives. Extracts taken from animal testes, ovaries, thyroids, and other organs were prescribed orally or were intravenously injected to treat mental illnesses. This article explores these treatments and argues that they are reflective of a broader interest in endocrinology and psychiatry in interwar Britain.

In the United States, the establishment of voluntary hospitals has been associated with attempts to normalize or stabilize the general population through the moralizing discourse and practice of psychiatry. For example, Elizabeth Lunbeck has argued that the foundation of the Boston Psychopathic Hospital opened up the asylum walls and extended psychiatric theory and practice into the domain of everyday life. Mind doctors were no longer purely concerned with severe pathology but began to scrutinize family relations, cultural traditions, work patterns, gender relations, child care, and sexual desire (Lunbeck, 1994). Similarly, the introduction of psychoanalytic theory and psychological services has been linked with new attempts to control and manage family units in the wake of large-scale industrialization. Focusing on the United Kingdom, Nikolas Rose has shown how the growth of professionals dealing in the field of “human relations,” such as social workers, marriage counselors, and juvenile court specialists, was intimately tied to economic transitions which placed the family at the centre of new governmental enterprises (Rose, 1985, 1999). Furthermore, Eli Zaretsky has argued that Freudian theory was as important to the new middle classes who worked within the new consumer economy of the 1920s and 1930s as Calvinism had been to the early pioneers of capitalism (Zaretsky, 2004). In other words, the expansion of voluntary psychological services have been directly associated with major social changes in the 1920s.

During the 1920s and 1930s, the relationship between medicine, psychiatry, neurology, psychology, and psychoanalysis was inherently fluid and disciplinary boundaries were only beginning to be drawn. Key figures in the history of British psychology such as William McDougall, Charles Myers, and William Rivers had all qualified in medicine. All three went on to influence the treatment of shell-shocked soldiers during the First World War using psychological methods, thereby challenging medical orthodoxy (Miller, 1996, p. 158). As John Forrester has demonstrated, the work of Myers and Rivers within the British Psychological Society (BPS) ensured an experimental and medical basis to the study of psychology in Britain. Drawing from the researches of Henry Head, Charles Sherrington, and others on the function of the brain and the nervous system, they argued that psychology was inseparable from medicine and, in 1919, formed a Medical Section to the BPS (Forrester, 2008, pp. 52–56). In addition, psychoanalysis, which had roots in the field of neurology, was achieving some acceptance within medical circles in Britain. The foundation of the British Psychoanalytical Society by the medically trained Ernest Jones in 1919 and the 1929 Report of the British Medical Association on Psychoanalysis secured this influence (King, 1991; Forrester, 2008). Although Edward Mapother was suspicious and sometimes overtly critical of psychoanalytic approaches (Jones, 2003), many of the doctors who regularly worked with patients at the Maudsley combined psychoanalytic theory with their knowledge of other contemporary sciences in order to develop mixed approaches.

Nineteenth century gendered approaches to insanity were still influential in Britain during the 1920s and 1930s. As Hilary Marland has pointed out, the diagnoses of “puerperal insanity,” “insanity of pregnancy,” and “lactational insanity” appeared in increasing numbers during the nineteenth century (Marland, 2003). These “female maladies,” as Elaine Showalter described them, were directly associated with the female body and doctors therefore began to link femininity to particular forms of insanity (Showalter, 1986, 1997). As has often been pointed out, nineteenth century gendered divisions in forms of madness reflected ideologies of sexual difference and also supported contemporary divisions of labor (e.g., Theriot, 1990). Men were more frequently diagnosed with creative forms of madness, criminal lunacy, and “masturbatory insanity” (Busfield, 1994). The First World War broke down some of the distinctions that had been drawn between forms of insanity but it also encouraged therapies which fused physical and psychological approaches (Shephard, 2001). When psychoanalytic therapies began to be used to treat patients in the United Kingdom, new forms of the gendered division of madness were created. Psychoanalytic theory prioritized sexual desire as the key determinant that defined an individual's psychological illness, rather than sexual function (Appignanesi & Forrester, 2005). At the Maudsley Hospital in the 1920s and 1930s, nineteenth century models of insanity were fused with new psychoanalytic approaches to form a model which interwove sexual desire and sexual function in the interpretation of an individual's problem. Maudsley doctors drew from Meyerian psychobiology but also developed their own approach.

The field of endocrine psychiatry and its relationship to the practice of psychiatric and psychological medicine in Britain has largely been neglected within historical study. Recently, Edward Shorter and Max Fink have begun to investigate the history of endocrine psychiatry although their work focuses on the 1970s (Shorter & Fink, 2010).^2^ Research conducted on Maudsley case records from the 1920s and 1930s suggests that endocrine treatments were often employed in conjunction with other psychological methods to reorient and redirect patients’ sexual and physical desires. This raises important questions about the role of glandular agents and endocrinological sciences in the uptake and expansion of psychological services in the inter-war period.

This article explores the alliance between endocrinology and the mental sciences, which developed at the Maudsley in the 1920s. It analyzes the impact that this had on the treatment of patients at the Hospital between the years 1923 and 1938. Although the focus of the article is specific, the broader implications of this phase in history are explored.

## Endocrinology, Psychology, and Psychiatry

The early history of endocrinology is intimately connected to the history of research and treatment of diseases of the human thyroid. As Thomas Schlich's recent work on organ transplantation has shown, the thyroid gland served as a paradigmatic organ for early human and animal experimentation on the function of glands and their role in disease processes. In 1873, William Gull of Guy's Hospital in London described the classic clinical picture of “myxedema,” which he regarded as a form of cretinism of adulthood, and the condition was named in 1878 by William Ord. The observation that patients with myxedema also exhibited neurological symptoms such as lack of perspiration, paresthesia, and slow or sluggish psychic functions initially led researchers to believe that myxedema was a neurological condition and that the atrophy of the thyroid was a secondary symptom caused by general vasoconstriction (Schlich, 2010, pp. 23–30). The study of human ovaries from the mid-nineteenth century had also led doctors to initially think that their function was regulated neurologically. The German gynecologist Rudolf Virchow first put forward this theory in 1848 and his work was later cited by many who claimed that the ovaries were responsible for a variety of illnesses in women (Sengoopta, 2006, pp. 11–15). It was only following surgical experimentation with the removal and transplantation of glandular organs and the injection of glandular substances that the endocrine system came to be regarded as functionally distinct from the nervous system. The work of the British experimental physiologist and neurosurgeon, Victor Horsley, and the Austrian gynecologist, Emil Knauer, were central in generating this new theory (Schlich, 2010, p. 38, pp. 87–88). The late-nineteenth saw an increase of experiments on sexual function, such as Josef Halban's experiments on castration and transplantation of ovaries in female baboons (Schlich, 2010, p. 89).

In 1889, the French physiologist Charles-Edouard Brown-Séquard (1817–1894) had published an article in the *Lancet* in which he described how he had injected preparations made from animal male sex glands into his own body. He claimed that these injections had led to improvements in his bodily functions and intellectual faculties (Borell, 1976, p. 312). In the same year, he drew attention to unpublished experiments in which women had been injected with the filtered juice of guinea-pigs’ ovaries to treat hysteria and other disorders of the uterus (Oudshoorn, 1990, p. 7). In 1891, the *British Medical Journal* published an article by George Murray, which demonstrated how he had injected emulsion from the thyroid gland of a sheep to treat a female patient with myxedema and this encouraged further experimentation with all types of glandular extracts (Murray, 1891). In 1893, the *New York Therapeutic Review*, the *Journal of the New York Pasteur Institute* published a three-part article entitled “Injections of Organic Fluids According to Professor Brown-Séquard's Method” which touted the use of organ extracts for the treatment of a range of illnesses including chorea, epilepsy, locomotor ataxia, and neurasthenia. It claimed that extract of pancreas could be used to treat diabetes, that extract of gray matter could treat neurasthenia, and that testicular extracts could be used to treat a range of diseases (Borell, 1976, p. 316). Chemical studies of thyroid and adrenal extracts followed these experiments. Adrenaline was isolated in 1901 and synthesized in 1905. Thyroxine was isolated later in 1914 and synthesized in 1927 (Hall & Glick, 1976: p. 231).

In 1905, the British physiologist, Ernest H. Starling, formulated the concept of a “hormone” to describe internal secretions from the glands or “chemical messengers” which were carried via the blood (Oudshoorn, 1994). He employed the concept for his Croonian lectures at the Royal College of Physicians on the “Chemical Correlation of the Functions of the Body” (Rolleston, 1936b, p. 2). Starling's descriptive concept stimulated further research into the relationship between glands and mental and physical functions. In 1907, the British physiologist Edward Sharpey-Schafer employed Starling's concept to argue that the testicle and ovary determined the development of secondary sexual characteristics through their production of hormones. He abandoned the theory that glandular functions were regulated by the nervous system and urged others to follow suit (Schlich, 2010, p. 89).

By the time that the Maudsley opened, theories of hormonal secretions or chemical messengers in the body were well established. In 1913, Artur Beidl's book *Innere Sekretion* contained a bibliography referencing over 8,000 articles on this topic. By 1925, more than 3,000 articles were published every year. In 1917, the Journal *Endocrinology* was established in Los Angeles. This was followed by the Italian *Endocrinologia e Patologia costituzionale* in 1922, the *Revue Française d'endocrinologie* in 1923, and the German *Endokrinologie* in 1928 (Rolleston, 1936a, p. 1).

William Siegfried Dawson was a senior member of the Maudsley's medical staff from 1923 to 1927. He had studied medicine at Trinity College, Oxford, and had served in the RAMC in East Africa and Egypt during the war. In 1920, he obtained the Diploma in Psychological Medicine from the Royal Colleges of Physicians and Surgeons and then joined the mental hospitals service of the LCC (Garton, 1993; Evans, Rahman, & Jones, 2008, p. 464). In 1924, he published *Aids to Psychiatry* in which he described how disorders of the thyroid gland, pituitary gland, suprarenal glands, pineal gland, and the gonads could cause mental symptoms ranging from depression, dementia, and irritability to sexual precocity, confusion, and excitement (Dawson, 1924, pp. 210–213). Dawson focused attention on the thyroid gland because research on this gland was the most developed. He described how a lack of thyroid production led patients to become “dull, defective in emotional reaction, and torpid” and to experience mental impairments of “perception, memory and ideation.” These patients also exhibited dry skin, loss of hair, as well as less active bodily functions, low temperature, and a “feeble” pulse. Conversely, in cases where there was an overproduction of thyroid, patients experienced “anxiety and fear” and were “apprehensive and irritable.” Dawson argued that in certain cases, the patient's restlessness progressed to “acute mania and death from profound toxemia and exhaustion.” In other cases “psychoses, such as melancholia or delusional states” could occur (Dawson, 1924, pp. 210–212). Drawing from this research, Dawson and other Maudsley doctors speculated that underproduction or overproduction of other glands could result in similar mental impairments or manias. For example, Dawson argued that lack of ovarian function led to “mental instability” and that disturbances of the ovaries during menstruation and the climacteric (menopause) led to “irritability and depression” (Dawson, 1924, p. 213).

Theories of the relationship between the functions of the female body and insanity were commonplace in the late-nineteenth century. Categories such as “hysterical insanity” and “puerperal insanity” were used by influential doctors such as David Skae, physician superintendent of the Royal Edinburgh Asylum, and Thomas Clouston, lecturer on mental diseases at the University of Edinburgh (Clouston, 1883). In 1873, Clouston gave the Morisonian Lectures on Insanity using the work of David Skae to identify 35 different types of insanity, just over a quarter of which were specific to women, namely “nymphomania,” “hysterical insanity,” “amenorrheal insanity,” “post connubial insanity,” “puerperal insanity,” “insanity of lactation,” “insanity of pregnancy,” “climacteric insanity,” and “ovarian insanity” (Skae & Clouston, 1873, p. 348). However, it was only during the 1920s—a crucial stage in the development of the disciplines of endocrinology, psychiatry, and psychoanalysis—that new theories began to develop in the UK concerning sex hormones and their relationship to the workings of the mind.

## Instincts, Drives, and Glandular Extracts

On February 10, 1922, Sir Frederick Mott gave a lecture to members of the British Medical Association entitled “The Reproductive Organs in Relation to Mental Disorders.” Mott was not only a critical figure in establishing the Maudsley Hospital, he was also a very influential lecturer and writer in physiology, pathology, and neurology and its relationship to clinical medicine. His early research work was carried out in Edward Sharpey-Schafer's laboratory at University College where he investigated cerebral cortex localization and conduction paths in the spinal cord among other topics. His later work at the Central Pathological Laboratory was critical in establishing the relationship between syphilitic infection and general paralysis of the insane (Anon., 1926; Sharpey-Schafer, 2004). Mott's contributions to neurology were wide-ranging, for example, he gave his Croonian lectures on the doctrine of the neurone and his Oliver Sharpey lectures on cerebrospinal fluid. He also wrote one volume of the Royal Society's Commission on Sleeping Sickness in 1906 (Anon., 1926, p. 1063). In 1920, Mott established a course of lectures and practical instruction in psychiatry at the Maudsley buildings, which then contained only a library and chemical and histological laboratories. The course was based on the Cambridge Diploma in Psychological Medicine and Mott employed senior British figures working in neurology and psychology such as Frederick Golla, a physician and neurologist who had previously studied with Victor Horsley at Queen Square Hospital (Hayward, 2004) and William McDougall, the eminent British psychologist and instinct theorist (Anon., 1920).

Mott maintained that individuals usually developed “psychoneuroses” and “psychoses” during periods when normal physiological changes were taking place in the body, for example, during adolescence, the “involutional” period in both sexes, or the puerperal period in women. Uniting psychoanalytic theory, instinct theory and the theory of hereditary transmission, Mott argued that mental breakdown during these critical developmental stages was “due to a failure of the vital impulse (*élan vital*) or libido of the psychoanalysts” to direct mental energy (Mott, 1922, p. 465). It was this failure that resulted in mental illness. Up until puberty, this vital impulse supposedly manifested itself “in the psycho-physical reactions for self-preservation by nutrition for the growth of the body and for its defense against injury” ( Mott, 1922, p. 465). After puberty, it was augmented and also directed toward sexual aims. It was during this time that it could be diverted or stunted, thereby causing mental illness. Speaking at the Charing Cross Hospital, London, in October, 1921, Mott had urged physiologists to use psychological theory in their work and *vice versa*. He argued that all human activities were rooted in primal instincts that were common to both men and animals and that he agreed with McDougall in his incorporation of Freudian psychoanalysis into British instinct theory (Anon., 1921).

In fact, Mott drew from both Freudian and Jungian theory on the “psychoneurosis,” under which category he included “epilepsy,” “neurasthenia,” and “hysteria.” He argued that “the bodily symptoms of many neuroses, such as hysteria and anxiety neurosis, could be cured by suggestion and persuasion” and that

The frequency with which psychoses and psychoneuroses follow emotional shock connected with the sex impulse, the character of the dreams and their interpretations, the nature of the hallucinations and delusions in a great number of the cases suggest an origin in excitement or repression of the sex instinct (Mott, 1922: p. 464).

When Mott wrote about the sex instinct, he was referring both to a psychological desire and to a biological instinct. Quoting Emil Kraepelin and his student Maurice Urstein, he pointed out that “most psychiatrists” posited a direct connection between “the insanity of adolescence” and “the sexual functions.” Both Kraepelin and Urstein had suggested that toxins from the sex glands might be a cause of mental disorder in adolescence. This work developed that of English asylum doctors such as Clouston who had identified an “insanity of pubescence” as early as the 1870s (Shorter, 1997, p. 105).

Mott sought to test these hypotheses by examining the sex organs of individuals who had developed mental disease. He argued that samples from patients with dementia praecox showed a “primary regressive atrophy of the reproductive organs.” Between 1919 and 1921, he conducted a series of studies published in the *British Medical Journal* and the *Journal of Mental Science* which explored the testes of 100 male patients with mental illness who had died in hospitals or asylums (Mott, 1919c, 1919b, 1919c, 1921). He also conducted examinations of the ovaries of women who had died in asylums, along with rates of reproduction in women who had previously been committed, concluding that many developed diseased ovaries and could not reproduce following the onset of mental illness (Mott, 1922). As a result of this research, Mott proposed that insanity was directly associated with physical changes in the vital organs of the body. In 1922, he claimed that in both men and women, it was likely that “all forms of insanity are associated with a tendency to failure of reproductive power” (Mott, 1922, p. 466).

Laboratory research in endocrinology was a core aspect of Maudsley research from its inception though it was not until the early 1920s that Mott and others fused this into a general theory of psychopathology. Following the outbreak of WW1, Mott moved the LCC Central Pathological Laboratory from Claybury Asylum to the Maudsley annex of King's in 1915, securing a number of Medical Research Committee grants to investigate the physiology of shell shock and other mental illnesses. The central nervous system, blood pressure, and endocrine functions all fell within his investigations (Mott, 1919e, pp. 16–22). At the end of the war, Mott estimated that 10 percent of servicemen admitted to 4th London General with neurasthenia “especially when trench warfare was taking place in 1915 and 1916 suffered with signs of hyperthyroidism” (Mott, 1919d, p. 709). Such findings encouraged him to seek an organic solution to shell shock and patients were given various medicines including pituitary and thyroid extract to treat their symptoms.

Hormonal treatments at the Maudsley were thus driven by a diverse range of medical theories from Mott's laboratory studies to gynecological research into the function of the human menstrual cycle and Freudian psychoanalysis. In addition, there was no clear division drawn between that which was mental and that which was physiological. As Mott had put it, “all psychical processes are subordinate to physiological processes, and all physiological processes are associated with, and dependent upon, oxidation processes” (Mott, 1922, p. 465). Aubrey Lewis, who joined the Maudsley in 1928 and served as clinical director from 1936 (Sheperd, 1975), took a similar view. Lewis had studied both medicine and anthropology before specializing in psychiatry. He trained with many critical figures in the history of psychiatry and neurology such as Macfie Campbell in Boston and Adolf Meyer at Johns Hopkins, Baltimore, and Gordon Holmes in Queen Square, London (Jones, 2003). This training led him to take an eclectic and open-minded approach to psychiatric illness In 1934, Lewis wrote that it was difficult to distinguish between “endogenous” and “reactive” depressions and psychoses because the human “organism” reacts both to internal “vegetative and endocrine” changes at the same time as it responds to environmental influences. Quoting Max Rosenfeld, director of the Psychiatric and Neurological Clinic in Rostock, Germany, he pointed out that

Since physical and psychic functions are fused into an indissoluble unity and not only in respect of sex there will also be possible in endogenous psychoses an active interplay between the psychic processes, the cerebral processes and the endocrines. It will often be difficult, perhaps impossible, to decide which processes are to be regarded as primary. (Rosenfeld, 1930, p. 375; Lewis, 1934)

The influence of Mott's laboratory research and Lewis's direction meant that glandular therapies were used at the Maudsley as one element of a wider system of treatment, which altered the patient's internal functions and nervous system. In addition, some patients were selected for psychotherapy to address their unconscious associations. Doctors sought to transform both the psychic and physical functions of their patients through this amalgamation of therapies.

## Controlling Sexuality

In order to analyze the treatments that doctors employed at the Maudsley during the interwar period, 50 patients treated with glandular extracts were selected at random from a larger database of patients treated between 1923 and 1927.^3^ Of the 50 randomly selected patients, 22 were treated with thyroid extract, 10 received thyro-ovarian extracts, and 10 were treated with parathyroid. The rest were treated with mixtures of hormones (see Table 1). Less than one-third of the cases were male. Based on surviving patient notes, the overall percentage of patients treated with glandular extracts from this period ranged from 5 to 10 percent of the total hospital population encompassing both inpatients and outpatients. However, inpatients were more likely to be prescribed glandular therapies than outpatients.

**Table 1:** A Random Sample of Maudsley Hospital Patients Treated with Glandular Extracts 1923–1927

	Female	Male
Thyroid	13	9
Thyro-ovarian	10	0
Parathyroid	7	3
Thyroid and thyro–pituitary–adrenaline	0	1
Thyroid and adrenaline	1	0
Thyroid and testicular extract	0	1
Thyroid and parathyroid	1	1
Thyro-ovarian and thyro-adrenaline	1	0
Thyro-ovarian, thyroid, and hormotone	1	0
Parathyroid and thyro–pituitary–adrenaline	1	0
Total	35	15

**Table 2:** Maudsley Patients Discharged in 1931 Treated with Glandular Extracts

	Female	Male
Thyroid	8	0
Thyro-ovarian	3	0
Ovarian	1	0
Hormotone	2	0
Parathyroid	2	0
Pituitary	1	0
Adrenaline	1	0
Pituitary–adrenaline	2	0
Thyroid, thyro-ovarian, and parathyroid	1	0
Total	20	0

**Table 3:** Maudsley Patients Discharged in 1935 Treated with Glandular Extracts

	Female	Male
Thyroid	19	4
Thyro-ovarian	3	0
Ovarian	1	0
Parathyroid	1	0
Thyroid gland removed	1	0
Total	25	4

Fewer patients were treated with glandular extracts in the 1930s. We therefore studied all cases treated with hormonal therapies from the archive of case records from 1931 to 1935. Of the 20 patients from the sample treated with hormonal therapies in 1931, all were women. Of the 29 patients treated in 1935, only four were men and they were all treated with thyroid extract.

There was a gradual shift away from the use of parathyroid, ovarian, adrenaline, and pituitary extracts from the 1920s to the 1930s. However, pure thyroid treatments remained popular. In addition, fewer men were treated with hormonal therapies in the 1930s. The only extract ever given to men in our sample from the 1930s was thyroid extract.

The Maudsley Hospital was the first publically supported mental hospital in the United Kingdom to provide institutional care for voluntary boarders. This meant that knowledge emanating from the LCC Pathological Laboratory could be directly tested on patients and the results could be observed and measured in a controlled environment. The doctors frequently discussed a patient's ability to “adapt” to their new circumstances, whether that followed a change in their job status, a death in the family, or a physiological change after puberty, menopause, or childbirth. Dr. Mary Barkas, who worked at the Maudsley from 1923 to 1927, described the hospital as a place of “shelter and refuge” which offered “complete protection and satisfaction of all needs.” Only in such an environment, she believed, could patients regress to an “antenatal state of freedom from stimulus and effort” from which doctors could begin the resocialization process. As she put it, the Maudsley:

supplies maternal nurses and paternal doctors; it puts him to bed – another symbolical regression to the intrauterine state – and drugs him into the infantile state of sleep where censorship is relaxed and the unconscious set free. It gradually retraces the child's development, trains him gently towards cleanliness and social decency, encourages sublimation of emotions into simple interests and occupations within a group of his equals in development. (Barkas, 1925, p. 335)

Barkas was originally from New Zealand but had studied medicine at St. Mary's Hospital, London and at the London School of Medicine for Women. She also had an MD in psychological medicine and had undergone intensive psychoanalytic training in Vienna (Dawson, 1959). She regularly prescribed glandular extracts as part of a wider treatment program encompassing psychoanalysis and institutional care.

When treating neuroses, Barkas sought to identify the original traumatic experience, which had led to a patient's conversion neurosis and then enable the patient to overcome this mental impasse. When working with psychotics, she drew from the work of John Rickman who was then working on *The Development of the Psycho-Analytical Theory of the Psychoses* (Rickman, 1928). Adopting Freudian concepts, Barkas maintained that psychotic individuals had regressed “from the reality principle to the pleasure principle’ and from ‘object relationships to auto-eroticism and pre-genital modes of gratification.” The goal of the doctor was to retrace the origins of these regressions and then to stop or invert them. However, in 1925, Barkas admitted that “we have not yet learnt how this backward journey can be stopped or reversed” and that further investigation was necessary into “the trends underlying character formation” as this would enable doctors to create “satisfactory sublimations” which prevented the patient from falling further into a regressive psychosis (Barkas, 1925, p. 335).

Barkas regularly employed glandular therapies in conjunction with talking cures. Another Maudsley physician who did this was Dr. Isabel Emslie Hutton, a Scottish physician who had written her thesis on the Wasserman reaction test for syphilis in the cerebrospinal fluid of the insane. Hutton was employed at the Maudsley as an honorary psychiatrist for seven years with no official post or salary because the LCC strictly forbade the employment of married women (Hutton, 1960, p. 218). In 1927, she was called up to serve on the British Medical Association's special committee to investigate psychoanalysis, which resulted in the 1929 Report (Hutton, 1960, pp. 240–246). Barkas and Hutton were the Maudsley's first female doctors and they both shared an interest in the relationship between psychological theories of the instincts and glandular functions.

All glandular therapies were considered to impact on either the sexual drive or the “vital impulse.” The prescription of “thyro-ovarian” or “ovarian” extract was perhaps the most overt way to influence a patient's sexuality and sexual behavior. Thyro-ovarian extracts were given to treat a range of mental states. For example, the 12 female patients from the 1923 to 1927 sample who were given thyro-ovarian treatments, either singularly or in combination, were diagnosed with “dementia praecox,” “spasmodic tic,” “anxiety state” and “scoliosis,” “obsessional anxiety,” “schizophrenia,” “depression with anxiety” and “anaemia,” “involutional melancholia,” “Dementia praecox (paranoid),” “depression with aural hallucinations” and “anaemia,” “paraphrenia” and two were diagnosed as “anxiety neurosis”. This diversity of diagnoses reflects the wide range of problems, which it was thought could be treated by redirecting and augmenting the patient's vital impulses.

The majority of extracts given at the Maudsley were pure glandular extracts taken from animal sources. Occasionally, patented products were prescribed. For example, “Hormotone,” formulated and marketed by G. W. Carnrick, was one preparation that was prescribed at the Maudsley. Described as “a physiological combination of tonic hormones derived from the thyroid, pituitary, ovary, testis, pancreas, and spleen in approximate physiological proportions” (Anon., 1913, p. 84), each tablet contained 1/20th grain each of desiccated thyroid and pituitary extract. In 1913, the product was analyzed at the Lancet Laboratory, where it was discovered that it contained a large amount of iodine. Carnick claimed that the drug had “a favourable effect on metabolism and oxidation, causing an increase in mental, nervous, and muscular activity” and that it was indicated in cases of “neurasthenia, premature senility and sexual neuroses” (Anon., 1913, p. 84). In 1936, Carnrick introduced a new formulation, Hormotone T, which combined 200 standardized international units of ovarian follicular hormone with the earlier formula (Anon., 1936). In the 1920s and 1930s, hormotone was given to both men and women, usually augmented with other glandular agents, though Hormotone T was aimed specifically at women.

Doctors explained glandular treatments through case narratives, which included both moral and medical reasons for using such therapies. Because some glandular extracts affected sexual function, these narratives usually contained information about sexual relationships. Most female patients who were prescribed glandular extracts in the 1920s and 1930s were also subjected to a thorough gynecological examination by Dr. William Gilliatt who commonly gave the diagnosis of an “infantile uterus.” Gilliatt had been appointed as assistant lecturer in obstetric and gynecological surgery at King's College Hospital in 1916 and he later became senior obstetric and gynecological surgeon from 1925 to 1946 (Peel, 2004). He worked closely with the Maudsley hospital in the assessment of female patients.

Case records from the 1923–1927 sample shed further light on how knowledge drawn from endocrinology, gynecology, and psychology was fused together in this period. For example, one 21-year-old woman had been admitted in April 1923 and treated with thyro-ovarian extracts, pure thyroid, and hormotone. Hutton diagnosed the woman with “compulsion neurosis” and claimed that this had been caused by the “condition of [her] sex organs.” She claimed that she had “an infantile uterus” and conducted a detailed study of the woman's nervous system. She found her deep reflexes to be “sluggish but equal” and also noted that she had a “timid rather anxious expression” on her face. Her circulatory system was found to be “poor” and she also had anemia. Hutton claimed that the most “important point[s]” concerning her condition were the fact that “she only started to menstruate last June” and that she had “underdeveloped” breasts and an “infantile uterus” (CFM 003.1125).^4^

The woman referred to her own illness as “the slow,” stating that it began at age 14 when she first began to work. She reported “pain in head” which occasionally moved to her neck. Hutton claimed that the woman's “compulsion neurosis” manifested itself in the development of “queer mannerisms,” for example “bobbing up and down for long before she finally sits down,” “hopping in and out of bed before she finally gets in,” and “touching things over and over again.” Hutton had a “fear it is an early dementia praecox” due to the woman's inability to free-associate and the presence of mannerisms. However, she believed that there was a possibility that the patient might improve “away from her mother” and she was therefore admitted. A passage from the typed case notes demonstrates the way that knowledge of endocrinology was fused with psychological theories:

Her mother's fussing at puberty about her amenorrhoea and warnings about men appeared to have made her regress towards childishness and she says she “wanted to remain a child”, cared for by and dependent upon her mother. Her fear of going out of her mind is associated with her amenorrhoea and with masturbation, which she feared had injured her. The compulsion to touch things, and her own body, are also connected with this (CFM 003.1125).

The patient was questioned in detail about her fantasies, dreams, sexual knowledge, and feelings toward marriage and pregnancy. This kind of questioning stimulated the production of discourse, which would not have been produced in many other contexts. The woman claimed that she did not want to marry but wanted to live with a female friend because she disliked the idea of sexual intercourse with men. She was encouraged to renounce this position. After eight months, the woman was reported to be “in good physical health and now menstruates regularly,” although “the obsessive doubt and slowness persist more than ever.” Although this woman had improved physically, she was not considered to be cured. Dawson recommended certification as he thought that “permanent care will evidently be needed ultimately.”

Other young women from the 1923 to 1927 sample treated with glandular extracts showed more favorable outcomes. One 21-year-old woman treated with thyro-ovarian extract had been assessed by William Moodie, the deputy medical superintendent who had trained in medicine, physiology, and pathology and psychology and had also served in the RAMC (Anon., 1960). Moodie diagnosed her with “anxiety state” and “scoliosis” and claimed that her illness had been caused by a “manic depressive type of personality,” “a love affair,” and “physical and emotional retarded development.” The woman had been suffering from crying fits and pain in her back, neck, and right arm for five months. She had also suffered from amenorrhea for three months and had only had light periods with long intervals since the age of 19. She was described by one Maudsley doctor as “a decidedly immature young woman” with “a history of some slight incontinence of urine” who “practiced self-abuse for many years without phantasy.”

Side-by-side with this account of the woman's intimate problems was an account of her economic status. She was noted to have grown up in “poor financial conditions” and to have developed only a “certain amount of intelligence” which enabled her to become a shorthand typist. The woman felt unable to commit to marriage, not feeling ready for housework, or other obligations. Summarizing the case, this doctor concluded that the:

Patient is quite definitely unbalanced and immature and seems to be passing through a phase resembling adolescence but the transit may be very slow. She states that she wishes she were a boy so that she could box, etc.

This woman was placed under the care of Dr, Rosalie Evelyn Lucas, a junior doctor who worked closely with William Moodie. Lucas began regular treatments of ultraviolet rays on the patient's back and also placed her on thyro-ovarian hormone. Shortly after this, Lucas reported that the woman had “less pain in [her] back” and was also “quite bright and cheerful and behaving normally.” She was also reported to have become “more stable emotionally” and to have gained “more insight than formerly” into her psychological problems and her social position. It was claimed that the glandular extracts had readjusted her endocrine balance, reaffirmed her womanhood, and she was discharged “recovered with approval” (CFM 004.1214).

Thyro-ovarian hormones were not only prescribed for young women who were experiencing problems with their sexual development but were also given to older women who were going through the menopause with the aim of stabilizing their mental state. Maudsley doctors maintained that if the lost hormones were replaced then the patient would return to their premenopausal mental state. For example, in 1923, one 51-year-old woman was diagnosed by Hutton with an “anxiety state” caused by the menopause. She was referred to Barkas who explored whether a glandular malfunction had influenced her beliefs and patterns of thought. Barkas created a narrative that wove together the patient's accounts of her physical condition with her past history, dreams, and associations. At one point, she reported on a detailed gynecological examination of the patient directly in-between accounts of her dreams as if the two types of facts held equal status:

She tells of more ‘shocks’ which she has had recently and which told on her nerves, one when she was driving with her brother and the horse ran away, the other when walking along a country lane she saw a man – whom she knew by sight – exposing himself. She complains of a slight discharge and losing occasionally and irregularly. Slight laxness of the pelvic floor, but no descent of uterus which is rather high up; ostium uteri externum and cervix normal to palpation.

Perhaps because the treatment was experimental, the patient was subjected to detailed examinations and the case material covered scores of pages. The patient was prescribed paraldehyde, salicin, and tribron along with daily exercise, a special diet and “high frequency” x-rays to head and spine. After these treatments proved unsuccessful, she was given thyroid extract and this was found to be extremely effective. The woman was reported to have steadily improved over several months of thyroid treatment as her anxiety subsided (CFM 005.298).

Other Maudsley doctors presented less elaborate case histories than Mary Barkas but nevertheless employed similar therapies for menopausal women. For example, in October, 1923, one 50-year-old female housewife was admitted under Alfred Petrie, who had previously worked as a medical officer at the London County Asylum in Bexley, Kent, and who had also served under the RAMC (Anon., 1962b). Petrie diagnosed the patient with “neurasthenia.” He noted that

She is rather evasive. Denies ‘voices’ but it seems likely that the noises are illusions if not hallucinations. She is inclined to think they are especially directed against her … She ascribes her illness primarily to the combination of menopause and air raids; its recent exacerbation to worry over her son (CFM 003.560).

The patient was given thyro-ovarian supplements together with bromides, caffeine, and high-frequency ultraviolet rays to her head and neck. No changes in her condition were reported and the staff regarded her as a problem female patient who was unlikely to improve and she was sent to a convalescent home. Many narratives contained within the case notes advanced the view that women should adopt normal domestic roles and that failure to do so was associated with mental suffering. In these cases, social adjustments were regarded as adequate cures for what were considered to be collectively social, moral, psychological, and physical problems.

The Maudsley theoretical approach was interdisciplinary in that it encompassed psychology, biology, evolutionary theory, and even the moral and social sciences, a union that was common in the writings of many British psychologists, anthropologists, and social scientists of the period such as McDougall and Rivers. However, Maudsley doctors sometimes prioritized pragmatism over theory and imposed their own views about normal behavior and beliefs when treating patients. There was very little interest in diagnostic categories or in recording symptoms as independent of a patient's individual history. While this method did not enable the doctors to amass statistical or other data on common symptoms and their recurrence, it did enable a system whereby familial histories were integrated with observed symptoms to gather a coherent picture of the individual patient's problem. Its limitations drew from the fact that many problems were found to be ultimately related to sexual or vital functions. The Maudsley psychoendocrinological model attempted to readjust patient's internal drives along with their minds. Although this often reinforced cultural norms, it also preserved each patient's individuality within the hospital setting.

## Male Patients

The theory and philosophy which supported the treatment of male patients with glandular extracts appeared to be less comprehensive than that which supported the treatment of women. Although testicular extracts were very occasionally given to male patients, they were not prescribed in the same way, or to the same degree, as ovarian extracts were prescribed to women. In most cases, glandular extracts were either given to men as shock treatments or were simply prescribed in small doses.

In the treatment of male patients, more emphasis was placed on testing intellectual abilities or tendencies toward hallucination and fantasy, rather than conducting detailed analyses of sexual drives and interests, although endocrine therapies were still employed to alter the sex drive. Mapother was very interested in psychological testing methods due to the close relationship between British psychology and psychiatry in the period. Writing in 1928, Mapother claimed that Charles Spearman's classic text on the measurement and analysis of mental functions, *The Abilities of Man,* should have the same impact on both normal and morbid psychology as Charles Darwin's *Origin of the Species* had on biology (Spearman, 1927; Miller, 1996). The use of psychological testing methods in the assessment and treatment of dementia was very evident in the male cases. At the Maudsley, the diagnoses of “dementia praecox” and “schizophrenia” were often given in cases where there was no evidence of hallucinations or delusions. In some cases, doctors translated dementia praecox literally as an “early dementia” which could manifest itself in various forms.

Three males from our 1923–1927 sample, aged between 21 and 24, were diagnosed with dementia praecox and/or schizophrenia and were given regular intravenous treatments with thyroid extract. In two cases, the onset was associated with excessive masturbation (CFM 015.776 and CFM 027.729). One of the patients believed that masturbation had damaged his mind so much that it was, in his words, “permanently ruined” however he continued to masturbate in the hospital and felt remorseful afterwards. In one case, it was reported that the patient had severe depression and that his “apathy and lack of interest are so marked that he has no real motive for living.” He was reported to have a “marked and definite affective loss, coupled with a great defect of emotion but unassociated with any cognitive defect.” The patient had “just that amount of insight into his condition to render him unhappy” but was “apparently not hallucinated,” had “no impulses,” and was “not suicidal.” In cases such as these, thyroid treatments were given to boost the nervous system and mental functions. This was hypothesized as having a secondary effect on sexual functions by stabilizing the patient's desire for uncomplicated or more socially acceptable sexual relations (CFM 015.776).

In late 1924, an “agitated garrulous man” was admitted under Dr. Horace Le Marquand for the duration of four months. Le Marquand was a junior doctor who had previously studied neurology and psychological medicine and had also served in the Royal Navy during the war. He developed a strong interest in endocrinology during his time at the Maudsley and later published widely on endocrine problems in children and adolescents (Le Marquand & Tozer, 1943; Anon., 1962a). Le Marquand diagnosed his patient with recurrent melancholia. The patient stated that he felt “depersonalised” and that his voice and limbs did not appear to be his own. He continuously asked himself questions such as “what has become of the innumerable dead?” Le Marquand described the patient to be “ego-centric and self-centred” as he talked of nothing but his own symptoms. Although most of the male patients were not questioned in detail about their sexual desires, there were instances where it was felt necessary to trace the patient's symptoms back to childhood using psychoanalytic techniques. In this case, the man was questioned about his early childhood and it was ascertained that he found out about “coitus” at the age of 13 and after this he felt unable to read aloud in front of his father or in class and felt terrible shame about his perceived lack of ability. These fears tormented him for almost two years until he had a complete “breakdown” at the age of 15 during which time he experienced his first feeling that “his body was something foreign to him and remote from his real personality.” The patient had recovered from this breakdown in adolescence but after a period of unemployment in his sixties, began to reexperience feelings of depersonalization. Mapother described him as “an elderly man of excellent education who has had considerable misfortune of late years.” Having identified “obsessional ideas concerning problems of time and space and general philosophy,” Mapother thought the prognosis was poor. The administration of bromides to promote sleep and regular doses of parathyroid extract had little effect (CFM 012.958).

The aim of parathyroid treatments was to stimulate the nervous system, a logic similar to that prompting the use of thyroid extract. However, parathyroid was used more often in the treatment of obsessional thought patterns and unfocused thoughts. For example, another male patient from the 1923 to 1927 sample who was treated with parathyroid was a 48-year-old man who was first diagnosed as “confusional” and then with “exhaustion psychosis.” Mapother noted that his memory was “fairly good” but went on to point out that

He remembers things back in childhood and also very recent events but is not so sure of happenings a few weeks back. Perception normal. Can execute simple arithmetical problems but rather inaccessible. He fails to do simple problems which would be solved by a normal adult. There is some evidence of considerable disorder of intellectual processes.

This patient was given regular parathyroid treatments and was reported to have improved gradually. After two months of inpatient treatment, the patient's confusion had cleared, his memory had improved, and his agitation had settled. The only problem that remained was that his thinking was still “childish” (CFM 014. 560).

## Thyroid Shock Treatment

Writing in 1924, Dawson argued that all manic depressive psychoses were “characterized by an excess of exaltation or depression which may be considered as an accentuation of normal variations of mood.” Under this category, Dawson included “melancholia” which, following Kraepelin, he regarded as one aspect of manic depressive psychosis. Dawson argued that melancholia could in some instances be treated with “hyperthyroid treatment” in which large doses of thyroid extract were given over a short period of time to cause an “acute illness” which stimulated the patient's nervous system. As Dawson put it, “during this period the patient looks ill and loses weight” but this was followed by a “remarkable improvement in the mental condition” (Dawson, 1924, p. 126).

In 1929, Henry Devine, Medical Superintendent at Holloway Sanatorium, Virginia Water, included a similar claim in his “Recent Advances in Psychiatry” (1929). He devoted a whole chapter devoted to shock therapy and claimed that

Glandular agents may be used, not only for their physiological effects in supplying what the organism lacks, but also as “shock” agents, with a view to the modification of the humoral-neuro-vegetative tonus. The gland extract in the latter case is not used for its specific effects, but as a form of protein therapy. It is thus that the good results obtained by some clinicians with injections of orchitic extract in psychotic females, and with ovarian extract in psychotic males, become explicable (Devine, 1929, p. 154).

Building on this observation, he argued that the hypothesis “that emotional trauma produces effects upon the organism similar to those occurring in protein and anaphylactic shock is one of considerable interest” (Devine, 1929, p155). There is no record of doctors giving ovarian extract to males or testicular extract to females in the Maudsley case-notes. However, there is evidence that doctors gave thyroid “shock” treatments in which large doses of thyroid gland would be administered over a day or a few days and then immediately ceased or reduced. These treatments appear to be precursors of insulin shock therapy, which became popular in the late 1930s and 1940s. This treatment had first been reported by Manfred Sakel in 1933 as a treatment for schizophrenia (Shorter, 1997, p. 209). Insulin shock therapy differed from thyroid shock treatments in that it produced convulsions though it drew from very similar principles.

In March 1924, one 36-year-old female patient was admitted under Le Marquand and diagnosed with “obsessional neurosis” and “melancholia.” The etiology of her condition was reported by Marquand to be “pregnancy.” The patient had previously suffered from what she described as “obsessions” shortly after she was married. She was treated by Dr. Millais Culpin with psychotherapy for a few months and Marquand claimed that she was “encouraged by him to have a baby.” Marquand's notes state that after she gave birth, she “began to say that she didn't know how to manage it and was not capable of doing it.” After this “she became depressed” and expressed “ideas of unworthiness.” Her sleep was frequently “disturbed by thoughts that she is going to smother her baby.” Doctors reported that her “feelings of unworthiness” were “not looked at in a rational light” but that “part of the reason for thinking she ought not to have had a child—her own mental instability—is a perfectly valid one” (CFM 003.543). The patient's sexual and marital relations were assessed in detail. It was reported that her “desire for sexual relations” had led her to marry a man whom she did not love. The assessment of the patient continued for several months during which time she was given regular sedatives, in particular paraldehyde.

Two months after admission, it was reported that the patient was “still very depressed and semi-stuperose,” that she had lost her appetite, and that she was only able to answer “no.” The patient also started to deny that she had a husband or a baby. One nurse also reported that the

Patient has been very depressed and stating that she wanted to die and has begged me to give her poison—she is “wicked” and is responsible for all the other patient's illnesses. She has removed her bedding since—denies her husband and states that her baby is dead.

As this patient did not show any signs of improvement following treatment of bed rest and sedatives, she was given a 10-day “intensive” course of thyroid treatment in which she was prescribed an incremental dosage of thyroid extract. She was given 10 grains of thyroid on the first day, and the dosage was then increased by five grains daily until it reached 30 grains. The dose was then gradually reduced. After this intense treatment, which lasted 10 days in total, the thyroid treatment was ceased entirely. She was then given 1/50 grain of strychnine, a stimulant, daily. Her progress was followed closely by the nurses.

One week after the thyroid shock treatment was over, the nurses reported that the patient “has shown great improvement the last week, is much brighter and shows an interest in her surroundings.” Le Marquand reported that the patient's pulse-rate had increased and that she later “became much brighter.” One month later, Mary Barkas noted that the

Improvement has continued steadily. She has gained weight and is active, cheerful and interested in her surroundings. She has been out on pass and visited her home and took a great interest in her baby.

The nurses also reported that the patient was “generally very bright and adapts herself easily in any circumstance.” She was discharged shortly afterwards with Barkas’ notes that she was “recovered,” “free from doubts,” and “completely well” apart from the fact that she had since developed amenorrhea (CFM 003.543). Although this new physical symptom was a result of the glandular treatments, the doctors were pleased that the patients’ mental state had improved and expected her periods to return in due course.

In 1924, one 52-year-old woman was admitted and diagnosed with “stress (following death of husband).” She was given bed rest for three months during which time she did not improve and was placed on “thyroid in large doses (‘shock’ therapy).” This woman was given doses of 20–60 grains daily for 10 days. After this she was reported by the same doctor to be “rather more agitated than usual, declaring that she can see herself becoming worse” though otherwise to have shown no change. She was later admitted to a convalescent home (CFM 003.630).

In some cases, “thyroid shock” treatments were administered repeatedly over the course of a few weeks. For example, in 1924, one 29-year-old male patient was admitted under Mapother and diagnosed with “dementia praecox” and “anxiety state” (CFM 020.119). Mapother reported the man's symptoms as “tension, depression, ‘nerves on edge’, slight clouding of consciousness” and “tremor of legs.”^5^ The patient had developed “shell shock” after three months in the trenches and was then employed behind the front line. After the war, he took over his father's business where he “did fairly well financially until about 15 months ago when loss of energy and defective concentration started.” Mapother stated that the patient had

Dreams of calamities, e.g. end of the world, that father is alive and insane. No battle dreams now or formerly. Stated by brother to have had two attacks at night of meaningful agitation (swearing and threatening his wife).

On admission, the patient was given one grain of thyroid extract which was then omitted. One week later he was given thyroid shock treatment. After the first treatment, Mapother reported that the patient had developed a “temperature of 100 with headache, pains in limbs and feeling of lassitude.” No symptoms or changes were reported after later treatments but after roughly 10 weeks, Mapother reported that the

Patient gave his notice yesterday and is leaving today. He is dissatisfied with the hospital and wants to try outside.

Variable responses to treatment are reported throughout the case-notes. Mapother and others did not conduct statistical trials to measure effectiveness. Nevertheless, the fact that thyroid and other glandular extracts continued to be administered suggests that doctors were convinced of their efficacy.

## Thyroid Research: Infants, Children, and Mental Development

In 1923–1924, the Medical Research Council awarded Frederick Mott a grant to investigate the basal metabolism of the insane and to conduct work on the iodine content of the thyroid gland (Jones & Rahman, 2009, p. 277).^6^ Following the work of Ord and Horsley, Mott claimed that, during gestation, infants received thyroid secretions from the mother which enabled the lower spinal neurons and the subcortical portion of the brain to develop. This hypothesis was supported by the observation that the thyroid gland of the mother produced more secretions during pregnancy, evidence which also supported the contention that women were more susceptible to endocrine disturbances. As Mott put it:

We have been looking at mind too much from the metaphysical point of view, and we have to realise that mind is dependent on the whole body, and the thyroid gland plays a very important part in mental development … In the case of myxoedema there is slowness of thought, slowness of action, and in many cases mental disorder, especially in women at the climacteric period, when the reproductive organs fail to produce their internal secretions, and there is a liability, in consequence, to a disturbance of the endocrine balance. (Mott, 1924, p. 522)

Reporting on a further analysis of 100 testes which he had conducted in conjunction with Isabel Hutton, Mott reported “a very marked change in the adrenal medulla” in cases where there was also a deficiency in the reproductive organs which manifested as insanity. He also reported similar changes in the pituitary in cases of insanity. He argued that thyroxin secreted by the thyroid gland was “essential for oxidation processes and neuronic function” (Mott, 1924, pp. 523–524). He called for “an intensive clinical and laboratory investigation of the whole reproductive endocrine glands and sympathetic system in the various types of mental disease” (Mott & Hutton, 1923).

The resignation of Mott as director of the Central Pathological Laboratory in March 1923 did not result in a new research strategy (Jones, Rahman, & Woolven, 2007, p. 358). Frederick Golla was appointed as his successor. In 1922, Golla had been invited to give the Royal College of Physicians’ Croonian lectures in which he discussed “The objective study of neurosis” (Hayward, 2004). He used this opportunity to criticize purely psychological approaches to mental illness, though pointed out that the psychoanalytic method had “given much valuable information in the realm of psychology” (Golla, 1921, p. 375). He believed that Mott had confirmed “the organic origin of dementia praecox,” and argued that “the profound metabolic disturbances” observed in manic depressive psychosis suggested that both illnesses were caused by a similar pathology (Golla, 1921, p. 379). Initially, Golla supported Mott's hypothesis that dementia praecox was a consequence of “a generalised degenerative anatomical change,” including both the adrenal and pituitary glands (Golla, 1929).

Laboratory work on the influence of the human thyroid on development and oxidation fed into clinical work via the treatment of children. Following the successful treatment of cretinism using thyroid extracts (Schlich, 2010, pp. 41–51), thyroid and other extracts were occasionally given to children to improve both their intellectual and physical development. Intelligence tests, based on those devised by Binet and Simon, correlated children's physical development with their mental development and also identified children whose minds had not developed and who therefore had to be classified as “mentally defective” institutionalized under the 1913 Mental Deficiency Act (Evans, Rahman, & Jones, 2008).

In 1927, one 16-year-old girl was admitted and given the diagnosis of “delinquent girl (mental defective).” She was also diagnosed with the physical condition of “hypothyroidism.” A thorough physical examination showed that the girl's hymen was “not ruptured” but that her retractions were “somewhat relaxed.” The girl claimed that she had only menstruated four times and that she had experienced a green discharge from her vagina. An intelligence test assessed her mental age at 12 years and 10 months with poor visual memory and arithmetic, fair auditory memory and ability to generalize, and good vocabulary, language ability, comprehension, and “mental control.” This intelligence level, together with her “sulky fits and wild outbursts of temper” and her “lazy spells” were regarded as a consequence of her physical condition. This was summarized as

Height and weight normal. Nutrition good. Frontal headache of the neuralgic type. Occasional vomiting ½ – ¾ hour after food. No cause found. Blood pressure distinctly low. Delayed puberty and infrequent menstruation. Suggest endocrine defect (CFM 028.271).

The girl was treated with thyroid extract and was reported to have steadily improved over the course of two months.

Similar styles of reasoning were used to treat more severe mental illnesses such as dementia praecox and mental deficiency. In 1925, one 17-year-old male was diagnosed by Petrie with the “mental disease” of “nervous instability” and the “bodily disease” of “stunted growth.” The notes stated that “mentally and physically he was always poor” and that his mental age was under 10 years. He was thought to have a “glandular defect” which was influencing his mental abilities and was suspected of having “mental deficiency.” The patient reported his own symptoms as characterized by feelings where “he does not quite know what he is doing and wanders about at times.” He was given thyroid treatments for a month and he gained 9 lbs of weight. Although not fit to be discharged, Maudsley doctors were pleased that he had developed the skills to work and he was eventually sent to a workhouse (CFM 008.238).

In 1924, one 16-year-old boy was admitted under Hutton and diagnosed with “dementia praecox (catatonia),” the etiology of which was understood to be “neurotic heredity” together with “adolescence” and a “congenital defect.” The boy was tested with a “psychogalvanometer,” an instrument which tested mental reactions by measuring changes in skin resistance after a voltage was applied to the skin. He was found to show no reaction at all. His symptoms were reported as “general feelings of lassitude” and he was found to have a “heavy and stiff gait,” slow “movements of expression,” “sweating on soles of feet,” bad articulation, and a facial expression showing constant “apathy.” The boy was given glandular extracts of thyroid and pituitary along with other sedatives such as bromides. He was also given “occupation therapy,” physical education, daily baths, and was “vibrated twice weekly.” These therapies were aimed at stimulating the nervous system, boosting the vital functions, and increasing intelligence. It was thought that this stimulation would improve the patient's insight and mental abilities enabling him to overcome his negativistic and apathetic mental state. The treatment was found to be partly successful in that the boy began to gain weight and to play and associate with others although he remained “apathetic” (CFM 009.888).

## Thyroid Treatments

In March, 1931, concerns were raised in the House of Lords over the use of insulin and thyroid extracts and whether they should be included as “poisons” under future legislation concerning the regulation of drugs. These discussions followed the Report of the Departmental Committee on the Poisons and Pharmacy Act which was published in 1930 (Willcox, 1939). In the resulting 1933 Pharmacy and poisons Act, they were not included as poisons but began to be more strictly regulated.^7^ It seems that with growing awareness of their potential dangers, Maudsley doctors refrained from using glandular extracts so freely. However, they did continue to use thyroid extract to treat melancholia and depression. Thyro-ovarian treatments were still given to women and more diverse glandular therapies were given in other illnesses, though usually only in very complex cases. Of the five patients from the 1931 sample who were treated with thyro-ovarian, ovarian, or mixed thyro-ovarian treatments, one was diagnosed with “hysteria with languid anergia,” one with “confusion and hyperthyroidism,” one with “schizophrenia” and “double aortic and mitral disease,” one with “confusional psychosis” and “septic mouth,” and one with “epiloia” or tuberous sclerosis causing the growth of benign tumors on the brain. Of the four who were given thyro-ovarian or ovarian treatments from the 1935 sample, one was tentatively diagnosed with “hysteria” or “dementia praecox,” one with “melancholia” and “endocrine disturbance” and two with “depression.”

Exactly half of all patients given thyroid extract in the 1923–1927 sample were diagnosed with forms of melancholia or depression (11 out of 22). In 1931, 62.5 percent of patients treated only with thyroid extract were diagnosed with melancholia or depression. In 1935, the percentage drops a little to 47.4 percent, although this can be explained by the fact that a larger majority of the patients were children (Evans, Rahman, & Jones, 2008). Children were sometimes treated with thyroid for conditions such as amentia (lack of mind) and mental deficiency and this was not the case with adults (e.g., CFM 151.645). The fact that Maudsley doctors consistently gave thyroid extract for the treatment of melancholia and depression throughout the 1920s and 1930s suggests that they considered it was an effective treatment for these conditions. If one looks further at the cases given other diagnoses, yet treated with thyroid, they are also often reported to have depressive symptoms (CFM 008.156). Many patients from the 1930s diagnosed with “dementia praecox” and “schizophrenia” were also described as having depressive and melancholic symptoms. Doctors frequently reported successes in treating depression and depressive states with pure thyroid gland.

As the 1930s progressed, further research was conducted on the use of thyroid to treat mental disorder. In 1933, Dr. M. A. Brazier, a researcher attached to Golla's laboratory, secured a grant from the Medical Research Council to investigate the “electrical impedance angle in disorders of the thyroid and psychoses” (Jones & Rahman, 2009, p. 278).^8^ In the following year the MRC granted Golla £50 a year toward the salary of Dr. Florence M. Grant to work on “the effects of the pituitary thyrotropic hormone in cases of involutional melancholia and other psychoses.”^9^ In 1929, R. G. Hoskins, Director of Research at The Memorial Foundation of Neuro-Endocrine Research, Worchester, Massachusetts, and F. H. Sleeper, assistant superintendent of the Worchester State Hospital, had reported that from a series of 80 patients presenting at their hospital with “dementia praecox,” 14 had shown evidence of thyroid deficiency. When treated with thyroid preparations, the patients were all reported to have shown marked mental improvements and four were reported to be completely cured (Hoskins & Sleeper, 1929b).

Building upon Pierre Janet's work on “psychasthenia,” Hoskins and Sleeper claimed that the patients suffered from a lack of nervous energy which resulted in a flight from reality. Janet's conception of the sense of reality, and how this could be lost, derived from a long line of research that he had conducted on patients in Le Havre from 1883 to 1889 and later the renowned Salpêtrière hospital in Paris. *La perte de la fonction du réel* was a disturbance of thought that was present in all psychasthenics, and which resulted from their inability to pay attention to different aspects of mental life. As Janet understood it, the function of reality was a synthesis of all psychological functions ranging from automatic functions taking place at the level of the nervous system up to complex thoughts and actions. The combination of attention to one's will or volonté in conjunction with an awareness of external reality formed the synthetic operation of presentification, the formation of the present moment in the mind. Janet drew from the work of the English sociologist and philosopher Herbert Spencer in developing a system of hierarchy of psychological functions, each of these functions having a different “coefficient of reality.” In Janet's view, an individual could potentially have a large amount of mental energy but be unable to use this within the higher mental functions. With high “psychological tension,” however, he could concentrate and unify psychological phenomena, thus, engaging in the highest function that of reality (Janet & Raymond, 1903; Ellenberger, 1970, pp. 61–137; Valsiner & Veer, 2000). Hoskins and Sleeper used this theory to explain the mental changes which followed from thyroid treatment arguing that vital drives and mental energy were altered through endocrine interventions which enabled patients to maintain a stable mental state (Hoskins & Sleeper, 1929a).

In 1938, Brazier published two articles in the *Journal of Mental Science* in conjunction with Russel Fraser, a Maudsley physician with a strong interest in endocrinology, and William Sargant, a Maudsley doctor and researcher who had trained with Edward Mapother and was a staunch advocate of physical treatments in psychiatry. These articles referenced Hoskins and Sleeper's thyroid treatments but critiqued their reliance on psychological theory as a justification for their effectiveness. They claimed that “numerous workers have experimented with thyroid treatment in mental disorder” but these treatments had not been measured effectively (Sargant, Fraser, & Brazier, 1938). Instead of relying upon psychological theory, they proposed recording electrical activity in the patient's body as a measure of the efficacy of thyroid in treating mental illnesses. They claimed that thyroid could be useful in a range of illnesses such as:

cases of recurrent katatonic excitement or stupor, cases of acute schizophrenia which exhibit a marked additional depressive component, and cases of depression which form part of a manic-depressive psychosis, or exhibit some depersonalisation, mild confusional features or retardation.

Their interest in the depressive aspects of schizophrenia and the psychotic aspects of depression help to explain why they considered it possible to treat schizophrenia and psychosis with thyroid extract.

In 1939, Golla took up a new position as director of the newly established Burden Neurological Institute (BNI) in Frenchay, Bristol. The institute was a private charity and Golla had considerable freedom to pursue his own research agenda. He recruited a team of young researchers (including Grey Walter who had worked at the Central Pathological Laboratory) specializing in electrophysiology and endocrinology (Hayward, 2004).

By the outbreak of the Second World War, endocrine treatments had become significantly less popular among Maudsley psychiatrists. In their textbook, *An Introduction to Physical Methods of Treatment in Psychiatry*, Sargant and Eliot Slater, who had served as a medical officer at the Maudsley Hospital from 1931 and worked at Sutton Emergency Hospital during the war, took a critical line (Sargant & Slater, 1944, pp. 128–134). They argued that hormones should not be used to treat involutional depression and that electro-convulsive therapy (ECT) was “very much more effective.” They also argued that the only group of patients who benefited from endocrinological treatment were cases of anxiety arising during the menopause (Sargant & Slater, 1944, p. 131). In part, their rejection of glandular therapies was conditioned by enthusiasm for two radical interventions: ECT and prefrontal leucotomy. In fact, it is Golla who holds the dubious distinction of authorizing the first prefrontal leucotomy performed in the United Kingdom, which took place at the Burden Institute in October 1941 (Golla, 1943). In addition, the Maudsley move away from endocrinological treatments was impacted by the fact that it was difficult to source quality hormones for the purposes of research following the outbreak of the Second World War. Maudsley doctors then began to focus on the neurochemistry of the brain rather than the study of hormones or “chemical messengers” which traversed the whole body before having an impact on the mind.

## Conclusion

The influence of British psychology and instinct theory, in particular the work of William McDougall, was clearly evident in the treatment of patients at the Maudsley Hospital during the 1920s and the early 1930s. Early Maudsley practitioners such as Dawson viewed the human mind as the by-product of more fundamental biological processes and reflex actions which drove the human organism. Dawson claimed that the human organism responded to environmental stimuli at four different levels. The “primitive, chemical level” which included the endocrine system; the “reflex action” which was driven by nervous response; the “instinctive action” where consciousness began to play a part; and the “volitional action” which was directed by the conscious mind and often conflicted with reactions from the lower levels (Dawson, 1924, pp. 5–6). In his research at the Maudsley laboratories, Mott also emphasized the need to consider the whole body when addressing psychiatric problems

We now generally recognise the brain as the seat of the psyche, but the functions of the mind are dependent upon the whole body and the harmonious interaction of all its parts.

Maudsley doctors’ interest in the use of glandular extracts to treat psychiatric disorder stemmed from an interest in the role of hormones as chemical messengers. They thought that if one could alter bodily reflexes and responses at their most primitive, chemical, level then this could also enable mental adjustment. Little was known of the exact chemical processes which the administration of organ extracts provoked within the body or brain, though interest into these chemical processes did grow in the Maudsley laboratories by the 1930s.

Focusing on the United States, Jack Pressman has explained the rise of lobotomy and intrusive medical interventions in psychiatric illness as a response to a growing urgency among psychiatric professionals to establish their discipline as a legitimate branch of medicine in the late 1930s. In Britain, Sargant, Slater, and others’ enthusiasm for such treatments was also driven by their desire to establish psychiatry as a medical science and to show that radical transformations were possible. In 1957, Sargant appeared in a documentary produced by the British Broadcasting Corporation (BBC) entitled “The Hurt Mind: Physical Treatments” which advocated the use of ECT, insulin shock, and lobotomy as effective methods of treatment and sought to present a positive public image for these interventions. This was the same year in which the Percy Report was published by the British Government which eventually led to the Mental Health Act 1959.

At the same time, laboratory research into psychiatric illness, which was less in the public eye and not so clearly directed by institutional changes, began to focus on the study of chemical and electrical processes in the brain, rather than the entire body. Again, this was part of the general trend to establish psychiatry as a specialist branch of science, distinct from psychology, biology, and general medicine. One research path which quickly gained momentum was neurochemistry. Juda Quastel, based at the research laboratories of Cardiff City Mental Hospital largely funded by the Rockefeller institute, investigated the effects of barbiturates on brain metabolism during the mid-1930s (Michael, 2010). At the same time, Derek Richter was also conducting similar work at the Biochemical Laboratory at Cambridge University. Along with Hermann Blasehko, he succeeded in purifying the enzyme that catalyzed the oxidation of epinephrine. They also conducted work on serotonin and dopamine. In 1938, Richter joined the research laboratories at the Maudsley began to conduct similar work with Golla (Gaull, 1996). Richter later claimed that it was Mapother who had brought together clinical psychiatrists and neuroscientists and that this had encouraged research in neurochemistry (Richter & Healy, 1995).

In many ways, the studies of brain metabolism which developed at the Maudsley from the late 1930s onwards were directly related to previous research in endocrinology. As neurochemical research increased in psychiatric circles, Otto Loewi's 1921 identification of a substance in the peripheral nervous system, later termed acetylcholine, grew in importance. As Richard Noll has pointed out, researchers initially used the terms “neurohormones” and “neurohumors” to describe internal secretions of nerve cells and the term “neuro-transmitter” did not begin to be used until the 1960s (Noll, 2006). Neurochemical researchers such as Richter always retained a clear interest in endocrinology. After moving to the Mill Hill Emergency Hospital during the war, Richter published a study of metabolic processes and endocrine disturbances in cases of insanity (Richter, 1944). R. E. Hemphill, director of clinical services at Bristol Mental Hospital, also continued to work on endocrine disturbances, publishing regularly in the *Journal of Mental Science* (Hemphill, 1944). In 1950, Richter edited a volume of essays on *Perspectives in Neuropsychiatry* that had been presented to Golla by past pupils and associates on his 70th birthday and which included a paper by Hemphill on endocrine disturbances (Richter, 1950). However, psychiatric research in biochemistry was increasingly restricted to the study of the brain as psychiatry became an increasingly specialized discipline in the postwar period.

British psychiatric researchers’ focus on the brain was driven by professional and institutional interests. Researchers such as McDougall already had quite developed theories of neurochemistry. McDougall's *Physiological Psychology* includes a sophisticated description of chemical transmissions throughout the nervous system in which he claimed that “every part of each neurone is irritable, i.e. is capable of responding to a stimulus with a katabolic change” and that “this katabolic change results in the conversion of chemical potential energy into free nervous energy” (McDougall, 1905 [1908], p. 31). However, researchers such as Mott did not develop these aspects of psychological theory and they did not influence psychiatric practice during the 1920s and 1930s. It was not until the mid-to-late 1930s, when psychiatric researchers targeted the brain as a site of research that neurochemistry was isolated from general mental science. It was also in this period that the use of glandular therapies at the Maudsley declined in practice and theories of glands and endocrinology fell out of general psychiatric discourse in Britain. This was replaced with the language of neurochemistry and new methods in psychiatric research which no longer drew from multiple sciences including instinct theory, general psychology and psychoanalysis, and the study of the chemical messengers of the reproductive, developing body. This fusion of research interests, which had been driven by both laboratory researchers and by doctors and nurses working on the wards, had enabled the formation of a glandular approach to mental disorder which has since been largely forgotten.^10^

## Footnotes

1 This was the finding of the United Kingdom's national Research Assessment Exercise 2008.

2 Today, endocrine psychiatry, or psychoneuroendocrinology, is described as a branch of psychiatric medicine which deals with the impact of hormonal changes on brain chemistry and psychological processes (Wolkowitz & Rothschild, 2003).

3 Bethlem Royal Hospital Archives Database: Maudsley Hospital 1923–1938, Bethlem Royal Hospital Archives and Museum (BRHAD: MH 1923–1938). The database contains the records of all surviving patient case records from the Maudsley hospital for the years: 1923–1927, 1928, 1931, and 1935.

4 BRHAD: MH 1923–1938. Reference numbers refer to the first number of each individual case record.

5 The symptom in double-quotes refers to the patient's own description of his problem.

6 See also: TNA, FD2/10, *Report of the Medical Research Council for the Year 1923–24*, London: HMSO (1924), p. 75.

7 The debate is recorded in the House of Lords Archive: Hansard *HL Deb 26 March 1931 vol 80 cc511–78*.

8 See also: TNA, FD2/20, *Report of the Medical Research Council for the year 1933–34*, London: HMSO (1935), p. 105.

9 TNA, FD6/3, Medical Research Council Minute Book, January 26, 1927 to June 19, 1936, October 26, 1934, it. 163.

10 There has recently been a growth of interest in the relationship between hormones and behavior, particularly in the fields of child psychology and neuroscience. For example, in *Prenatal Testosterone in Mind* (2004), Baron-Cohen et al. argued that prenatal hormonal levels can be directly related to the development of particular behavioral traits in children. (Baron-Cohen, Lutchmaya, & Knickmeyer, 2004, preface). This research builds upon animal studies in endocrinology and behavior and brings them closer to the fields of human psychology and psychiatry. Other researchers have claimed to have identified cognitive differences in animals caused by hormonal changes and have created animal models for well-known psychiatric problems such as depression and schizophrenia, for example, the work of Knut Larsson (Ågmo & Vega Matuszczyk, 2003). Although researchers such as Cohen have focused on hormones and sexual difference; other neuroendocrinologists have examined nonsex-specific topics such as the endocrinology of stress responses (Romero & Butler, 2007). Journals such as *Frontiers in Neuroendocrinology* (1969–present) and *Hormones and Behavior* (1969–present) have distributed this work which is increasingly being integrated into the broader field of neuroscience. Several recent studies have shown that the isolated thyroid hormone, thyroxin, appears to augment antidepressants in resistant depression (Aronson et al., 1996; Joffe & Sokolov, 2000). Furthermore, recent research in neuroscience suggests that dopamine and serotonin levels are correlated with pituitary–thyroid state (Strawn et al., 2004). Finally, controversial new research is suggesting that estradiol can also augment antidepressants in the treatment of female depression (Morgan et al., 2005).
